# Pulmonary Tuberculosis in General Practice

**Published:** 1917-04

**Authors:** 


### Reviews of Books.

**Pulmonary Tuberculosis in General Practice.** By HALLIDAY G. SUTHERLAND, M.D. Edin. Pp. xiv. 290. London: Cassell & Co. 1916. Price .

As the title indicates, this book gives the modern conception of pulmonary tuberculosis as a systemic disease, with an account of clinical and biological methods of diagnosis and the rational treatment of the malady. One application of the facts is of considerable practical importance to general practitioners. " In practically every household, rich or poor, with a single case of aggressive or chronic infectious pulmonary tuberculosis, there are other cases waiting to be recognised. When the disease has been diagnosed in one member of a family all the others should be carefully examined. It is well to detect lessons when these are most amenable to treatment, and when that treatment may be represented by the simple rules of the physiological life."

